# Challenging the nexus of power: The gaming dilemma of collaboration between government and enterprises in environmental management

**DOI:** 10.1016/j.heliyon.2023.e23472

**Published:** 2023-12-10

**Authors:** Feng Cao, Ling Zhang, Weiyun Wu, Sijia Han, Zhaocheng Wu, Yihan Wu

**Affiliations:** a School of Economics and Management, Fuzhou University, Fuzhou, China; b School of Social Development, Nanjing Normal University, Nanjing, China; c China Construction Bank Wenzhou Branch, Wenzhou, China

**Keywords:** Environmental governance, Mobilized governance, Government-enterprise collusion, Evolutionary game

## Abstract

This study presents a novel approach to analysing environmental governance by Chinese governments through the lens of a tripartite evolutionary game model. The key novelty of our research lies in the explicit incorporation of a reputation mechanism into the evolutionary game analysis, which significantly influences government decision-making, environmental governance strategies, and the dynamics of the relationship between government and polluting enterprises. By assessing the marginal effects of conventional governance and mobilization-based governance on the environmental mechanism, as well as the collusion behaviours between government and polluting enterprises, our study sheds light on previously unexplored aspects of environmental governance. Our findings indicate that the reputation mechanism plays a crucial role within the evolutionary game system of environmental governance, exerting a substantial impact on government decision-making. Furthermore, we demonstrate that policy interventions, such as increasing the cost of penalties under regulatory policies, can encourage compliance strategies among enterprises. Additionally, our research highlights the high sensitivity of reputation signals towards local government's environmental governance strategies, directly impacting their credibility and influencing the adoption of proactive environmental governance strategies. Moreover, we emphasize the role of the central environmental inspection system as a means to facilitate the transmission of environmental governance pressures between central and local governments, ultimately fostering a green development concept and promoting coordinated development between the economy and ecological civilization. Overall, this study provides valuable insights into the intricacies of environmental governance in China and underscores the importance of reputation mechanisms and policy interventions in promoting sustainable development.

## 1 Introduction

China has experienced rapid economic growth in the wake of its reform and opening-up policies. However, this journey towards prosperity has been accompanied by an undeniable reality: persistent environmental degradation. In 2020, Yale University's Environmental Performance Index revealed that China's overall score fell significantly below the global average, ranking a mere 120th out of 180 participating nations [1]. Alarming as it may be, this is not an isolated issue. China grapples with prevalent environmental pollution, which frequently gives rise to social conflicts.

Several statistical data indicate a disquieting trend. Chinese environmental conflicts have surged at an average annual rate of 25 % since 1997 [2,3]. The ramifications of such pollution are far-reaching, hampering not only China's pursuit of sustainable economic development but also posing substantial threats to social order. Recognizing the gravity of this situation, China's Central Government has turned their attention to this urgent matter. The 14th Five-Year Plan unequivocally calls for expediting the advancement of green and low-carbon development, continuously enhancing environmental quality, strengthening the central ecological and environmental protection inspection system, and intensifying the battle against pollution. In light of these circumstances, it becomes imperative to conduct an inquiry into the current mechanism of environmental governance and its attendant repercussions.

In China, two primary approaches have emerged to compel local governments in fulfilling their responsibilities for environmental protection: strengthening conventional governance and implementing mobilized governance [4,5]. Conventional governance, deeply ingrained in China's compartmentalized governance structure, manifests through the division of labor for ecological goals, the integration of environmental criteria into official performance evaluations, and the establishment of vertical systems for environmental supervision. Conventional governance relies on the transmission of environmental governance pressure from higher levels of authority down through the hierarchical system. It also entails incorporating principles of ecological civilization within the metrics that evaluate the performance of local officials. However, despite the existence of top-level designs and financial policies, the effectiveness of environmental governance falls short of expectations due to disparities in economic development and varying perceptions among officials. Confronted with immense pressures surrounding environmental protection, certain local governments have encountered dysfunctional environmental governance practices, including collusion between government and enterprises, a lack of adequate enforcement of environmental laws, and prevalent instances of inaction in environmental governance [5,6]. These challenges stem from the difficulty of establishing cross-regional environmental governance mechanisms and local deviations in implementing national environmental protection policies. Consequently, local governments find themselves compelled to engage in negotiations regarding environmental governance issues. Furthermore, long-term frameworks for local environmental governance remain inadequate, and the absence of meaningful participation from the general public exacerbates the risk of encountering the “tragedy of the commons”. In light of these factors, it becomes crucial to address both the systemic and participatory shortcomings within local environmental governance.

In the realm of environmental governance, scholarly focus has gravitated towards several key areas. Primarily, attention has been directed towards the intricate interplay between the central government and local governments [[7], [8], [9], [10], [11], [12]]. Through the lens of organizational control, imperative adjustments are urgently required in terms of reallocating responsibilities, fine-tuning incentive mechanisms, and fostering transparent information channels within the vertical regulatory framework of environmental governance. It is worth noting that power dynamics pertaining to environmental governance issues exhibit regional disparities, with diminished authority typically observed at the local level, that is, an inverse correlation to the levels of economic development and information asymmetry [13,14]. Some local governments still grapple with fully embracing the vision set forth by the central government for the construction of an ecological civilization, often prioritizing economic growth over environmental preservation. Regrettably, higher authorities frequently adopt flawed governance practices, characterized by campaign-style approaches, data fabrication, and other systemic deficiencies when assessing environmental indicators.

The second focal point lies in unraveling the intricate relationship between governmental bodies and enterprises [6,[15], [16], [17], [18], [19]]. Under China's administrative system, environmental governance assumes a distinct territorial character, with local governments serving as the driving force behind policy implementation at the grassroots level. This dynamic consequently engenders pronounced variations across territories in the effectiveness of central environmental policy implementation [10,20]. Presently, local governments are compelled to prioritize economic growth over competing development objectives such as environmental protection due to the prevalence of promotion tournaments [20,21]. Recent studies have substantiated the inclination of local governments to “sacrifice the environment for economic growth” when provided with opportunities to partake in promotion tournaments. Absent a robust and effective regulatory framework, this manifests in the relaxation of environmental protection constraints imposed upon enterprises operating within their jurisdictions, thus clandestinely facilitating heightened production levels among polluting industries [3,12,21,22]. Unfortunately, the collusion between local governments and polluting enterprises engenders a mutually beneficial, yet deleterious scenario for both parties—a situation that contradicts the overarching aspirations of the central government and society at large. Consequently, despite burgeoning investments in environmental management with each passing year, the resultant outcomes fall frustratingly short of expectations. Scholars have expressed profound concerns regarding the pivotal role played by the government-enterprise relationship in environmental governance, contending that the corrupt practices of local officials wield substantial influence over the effectiveness thereof, while restraining collusive tendencies can effectively mitigate regional environmental pollution [6,11,23].

Thirdly, the development of an all-encompassing environmental governance framework assumes paramount importance. Presently, China's environmental governance system encompasses conventional governance models alongside mobilized governance mechanisms, the latter of which endeavors to address the inadequacies inherent within traditional approaches. Mobilized governance hinges upon the implementation of rigorous disciplinary and incentive measures designed to catalyze proactive environmental enhancement efforts on the part of local officials through administrative directives and other coercive means [24,25]. Illustrative manifestations of mobilized governance include the environmental interview system and the environmental inspection mechanism. By means of the environmental interview system, competent authorities such as the Ministry of Ecology and Environment reinforce the primary responsibilities of local governments pertaining to environmental governance, through direct engagement with local officials, thereby curbing strategic emission reduction tactics often employed by these enterprises.

The central government employs various mechanisms to address the shortcomings of conventional governance, and one such mechanism is the system of environmental protection inspectors. This system utilizes top-down political mobilization to compel local governments to fulfill their environmental protection obligations. Concurrently, central environmental protection departments establish inspection teams to oversee environmental governance at the local level. Since the deployment of the first batch of central environmental inspection teams in July 2016, China has successfully completed two rounds of central ecological and environmental inspections, resulting in the resolution of over 160,000 ecological and environmental issues. Additionally, 262 illustrative cases have been made public, highlighting the significance of sustainable development concerns in 33.2 % of the cases and exposing formalities, bureaucracy, and instances of falsification in 18.3 % of the cases [26,27]. These environmental protection inspectors play a vital role in pushing local governments to abide by environmental protection regulations, circumventing the inherent limitations of vertical management imposed by administrative divisions. However, it remains essential to examine whether the system of environmental protection inspectors possesses legal authority. Moreover, the more important question is how to streamline potential collaboration between these enterprises to achieve more effective environmental control, and whether integrating reputation signals into the environmental governance mechanism can bolster local governments’ motivation.

Accordingly, this study employs an evolutionary game analysis to scrutinize the origins, underlying motives, and operational rules governing local governments’ predicaments in environmental governance. Additionally, it explores the intrinsic connections between collusion among government and polluting enterprises and the environmental protection inspection system. By introducing a reputation mechanism into the environmental governance framework, this study puts forth novel ideas to surmount the dilemma posed by government-enterprise collusion. The main contributions and innovations of this study lie in its comprehensive integration of the central government, local governments, and polluting enterprises within a unified analytical framework. This novel approach enables a more precise assessment of the marginal impact of government-corporate collusion on environmental governance. Furthermore, in order to enhance and complement previous research findings on the correlation between corporate collusion and environmental pollution, this study delves into the marginal influence of reputation signals on the phenomenon of corporate-government cooperation in environmental governance. Overall, the method of employing a tripartite evolutionary game model providing a robust analytical framework that captures the complexities, dynamics, and strategic considerations inherent in environmental decision-making processes, thereby offering valuable insights for both scholarly research and practical policy implications.

## 2 Game analysis of the evolution of environmental governance considering reputation mechanisms

The proposed game model aims to analyze the evolution of environmental governance by considering reputation mechanisms. It incorporates three key players: the central government, local governments, and polluting enterprises. The model seeks to explore the underlying causes of collusion between the government and enterprises while examining the marginal effects of reputation signals on the strategic decisions of both the government and polluting enterprises. Additionally, the model takes into account the operational logic of environmental governance within China's institutional context.

The fundamental assumptions of the model are as follows: 1) the game involves interactions and competitions between the central government and local governments, as well as between local governments and polluting enterprises. Each party has its own interests and objectives; 2) the ultimate goal of the game is to maximize the overall interests of all three parties. However, due to diverging interests, decision-making is decentralized among the parties; 3) the central government and polluting enterprises are considered finite rational enterprises capable of learning and imitation. They can adapt their plans and strategies based on changing circumstances.

### 2.1 The strategic choices of three parties

Firstly, the strategic choices of the central government encompass either “conventional governance” or “environmental supervision”. The former entails the traditional hierarchical approach to cascade environmental protection pressures from top to bottom, with relatively limited constraints. The latter employs compulsory measures such as inspection and supervision to compel polluting enterprises or local governments to reduce pollutant emissions, thus eradicating the occurrence of false governance by local governments and jurisdictional enterprises.

The strategic choices of local governments are divided into “active governance” or “collusion with polluting enterprises”. The strategy of “active governance” is adopted when local officials accurately weigh the relationship between economic development and environmental governance. To avoid being subject to “a veto” caused by reports from local residents or higher-level accountability, they actively fulfill their environmental governance responsibilities. This includes urging polluting enterprises to transform their production methods towards green alternatives in order to reduce environmental pollution and achieve genuine environmental governance in their jurisdiction. The strategy of “collusion with polluting enterprises” refers to refers to the phenomenon where certain local governments engage in partnerships or collaborations with polluting enterprises. Some local governments find it difficult to abandon the development mindset of “trading the environment for economic growth”. They still prioritize economic development and disregard regional environmental benefits. Faced with evaluations from higher-level governments, local officials tend to adopt strategies of collusion with polluting enterprises, thereby resulting in phenomena such as “symbolic environmental governance” and “false environmental governance”.

The strategies employed by polluting enterprises include “green production” or “seeking collusion”. When faced with serious government accountability, polluting enterprises actively embrace their social responsibilities and promote green production. However, when the cost of environmental pollution penalties is low and the cost of transitioning to green production is high, polluting enterprises, driven by factors such as production costs and return on investment, neglect the pollution caused by their operations on the local environment. They actively seek “consensus” with local governments to evade their environmental obligations.

Hence, in the tripartite game of environmental governance, the strategic combination of the central government consists of [environmental supervision, conventional governance], the strategic combination of local governments consists of [active governance, collusion with polluting enterprises], and the strategic combination of polluting enterprises consists of [green production, seeking collusion]. Let the probability of the central government conducting environmental supervision be denoted as ꭓ, the probability of local governments actively governing environmental pollution be denoted as y, and the probability of polluting enterprises promoting green production be denoted as z. In addition, the detailed variables of the model are set out in Table 1.

**Table 1:** Description of symbols.

Symbols	Description of the symbols
$G_{c1}$	Costs incurred by the central government during environmental supervision
$G_{R1}$	Subsidies granted by the central government to local governments during environmental supervision
$G_{R2}$	The central government offers incentives to polluting enterprises for embracing green production during environmental supervision
$G_{s1}$	The central government will impose punitive measures against the local governments for collusion with polluting enterprises during environmental supervision
$G_{s2}$	The central government penalizes polluting enterprises who opt for the strategy of seeking collusion during environmental supervision
$G_{s3}$	Polluting enterprises that embrace green production can garner favourable word-of-mouth benefits for the central government
$G_{s4}$	The central government receives high accolades from the society and experiences enhanced reputation due to its evaluations during environmental supervision
$G_{c2}$	When the central government pursues the strategy of conventional governance, its reputation suffers due to inadequate oversight
$G_{c3}$	Polluting enterprises resort to collusion when their own development falters and results in reduced government revenue.
$D_{c1}$	The strategy of active governance adopted by the local government necessitate substantial investments in human, material, and financial resources
$D_{c2}$	The fundamental costs borne by the local governments when entering into the strategy of collusion with enterprises
$D_{s1}$	The reputational advantages that can be gained when the local governments actively engage in governance
$D_{s2}$	The local governments choose to collude with polluting enterprises to obtain additional revenue through free-riding behaviours
$D_{c3}$	The society loses faith in the government as a consequence of collusion between local governments and polluting enterprises
$Q_{s1}$	The reputational value that can be generated by polluting enterprises choosing green production
$Q_{s2}$	The benefits for polluting enterprises when they choose green production and collude with the local governments
$Q_{c1}$	The extra costs associated with implementing green production for polluting enterprises when colluding with local governments
$Q_{s3}$	The drawback encountered by polluting enterprises when choose to the strategy of seeking collusion
$Q_{c2}$	The impact on corporate integrity when polluting enterprises choose to the strategy of seeking collusion

### 2.2 Model construction

According to the above assumptions, the central government, local government and polluting enterprises continuously adjust their respective behavioral strategies in the process of environmental management, and their benefit matrix is shown in Table 2.

**Table 2:** The benefit matrix.

The Local Government	Polluting Enterprises	The Central Government	
		Environmental Supervision x	Conventional Governance 1−x
Active Governance y	Green Production z	$G_{s4}+G_{s3}-G_{c1}-G_{R1}-G_{R2}$	$G_{s4}+G_{s2}-G_{c1}-G_{R1}-G_{c3}$
		$D_{s1}+G_{R1}-D_{c1}$	$G_{R1}-D_{c1}$
		$Q_{s1}+G_{R2}$	$Q_{s3}-Q_{c2}-G_{s2}$
	Seeking Collusion 1−z	$G_{s4}+G_{s3}+G_{s1}-G_{c1}-G_{R1}-G_{R2}$	$G_{s4}+G_{s2}+G_{s1}-G_{c1}-G_{R1}-G_{c3}$
		$D_{s2}+G_{R1}-D_{c2}-G_{s1}-D_{c3}$	$G_{R1}-D_{c2}-G_{s1}$
		$Q_{s2}+G_{R2}-Q_{c1}$	$Q_{s3}-Q_{c2}-G_{s2}$
Collusion with Polluting Enterprises 1−y	Green Production z	$G_{s3}-G_{c2}$	$-G_{c3}-G_{c2}$
		$D_{s1}-D_{c1}$	$-D_{c1}$
		$Q_{s1}$	$Q_{s3}-Q_{c2}$
	Seeking Collusion 1−z	$G_{s3}-G_{c2}$	$-G_{c3}-G_{c2}$
		$D_{s2}-D_{c2}-D_{c3}$	$-D_{c2}$
		$Q_{s2}-Q_{c1}$	$Q_{s3}-Q_{c2}$

#### 2.2.1 The central government's expected return and replication dynamic equations

Equation (1) describes the central government expects to gain benefits by implementing environmental supervision.

(1) $$G_{1}=yz\left(G_{s4}+G_{s3}-G_{c1}-G_{R1}-G_{R2}\right)+\left(1-y\right)z\left(G_{s4}+G_{s3}+G_{s1}-G_{c1}-G_{R1}-G_{R2}\right)+y\left(1-z\right)\left(G_{s4}+G_{s2}-G_{c1}-G_{R1}-G_{c3}\right)+\left(1-y\right)\left(1-z\right)\left(G_{s4}+G_{s2}+G_{s1}-G_{c1}-G_{R1}-G_{c3}\right)$$

The expected benefits of the central government's choice of conventional governance are $G_{2}$, as assumed in equation (2).

(2) $$G_{2}=yz\left(G_{s3}-G_{c2}\right)+\left(1-y\right)z\left(G_{s3}-G_{c2}\right)+y\left(1-z\right)\left(-G_{c3}-G_{c2}\right)+\left(1-y\right)\left(1-z\right)\left(-G_{c3}-G_{c2}\right)$$

The average expected return to the central government is $\bar{G}$, as assumed in equation (3).

(3) $$\bar{G}=xG_{1}+\left(1-x\right)G_{2}$$

The replication dynamic equation for the central government's choice of environmental supervision is shown in equation (4).

(4) $$F\left(x\right)=\frac{d\left(x\right)}{d\left(t\right)}=x\left(G_{1}-\bar{G}\right)=x\left(1-x\right)\left(G_{1}-G_{2}\right)=x\left(1-x\right)\left[-yG_{s1}-z\left(G_{R2}+G_{s2}\right)+\left(G_{s4}+G_{c2}+G_{s2}+G_{s1}-G_{c1}-G_{R1}\right)\right]$$

#### 2.2.2 The local government's expected returns and replication dynamic equations

Equation (5) describes the local governments expect to gain benefits by choosing the strategy of active governance.

(5) $$Q_{1}=xz\left(D_{s1}+G_{R1}-D_{c1}\right)+\left(1-x\right)z\left(D_{s1}-D_{c1}\right)+x\left(1-z\right)\left(G_{R1}-D_{c1}\right)+\left(1-x\right)\left(1-z\right)\left(-D_{c1}\right)$$

The expected benefits for the local governments opting for the strategy of collusion with polluting enterprises are $Q_{2}$, as assumed in equation (6).

(6) $$Q_{2}=xz\left(D_{s2}+G_{R1}-D_{c2}-G_{s1}-D_{c3}\right)+\left(1-x\right)z\left(D_{s2}-D_{c2}-D_{c3}\right)+x\left(1-z\right)\left(G_{R1}-D_{c2}-G_{s1}\right)+\left(1-x\right)\left(1-z\right)\left(-D_{c2}\right)$$

The average expected return to the local governments is $\bar{Q}$, as assumed in equation (7).

(7) $$\bar{Q}=yQ_{1}+\left(1-y\right)Q_{2}$$

The dynamic equation for the local governments to choose the strategy of active governance replication is shown in equation (8).

(8) $$F\left(y\right)=\frac{d\left(y\right)}{d\left(t\right)}=y\left(Q_{1}-\bar{Q}\right)=y\left(1-y\right)\left(Q_{1}-Q_{2}\right)=y\left(1-y\right)\left[xG_{s1}+z\left(D_{s1}+D_{c3}-D_{s2}\right)+D_{c2}-D_{c1}\right]$$

#### 2.2.3 Polluting enterprises expected returns and replication dynamics equation

Equation (9) describes the polluting enterprises expect to gain benefits by choosing the strategy of green production.

(9) $$W_{1}=xy\left(Q_{s1}+G_{R2}\right)+x\left(1-y\right)\left(Q_{s2}+G_{R2}-Q_{c1}\right)+\left(1-x\right)y\left(Q_{s1}\right)+\left(1-x\right)\left(1-y\right)\left(Q_{s2}-Q_{c1}\right)$$

The expected benefits for polluting enterprises choosing the strategy of seeking collusion are $W_{2}$, as assumed in equation (10).

(10) $$W_{2}=xy\left(Q_{s3}-Q_{c2}-G_{s2}\right)+x\left(1-y\right)\left(Q_{s3}-Q_{c2}-G_{s2}\right)+\left(1-x\right)y\left(Q_{s3}-Q_{c2}\right)+\left(1-x\right)\left(1-y\right)\left(Q_{s3}-Q_{c2}\right)$$

The average expected return for polluting enterprises is $\bar{W}$ , as assumed in equation (11).

(11) $$\bar{W}=zW_{1}+\left(1-z\right)W_{2}$$

The replication dynamics equation for polluting enterprises choosing the strategy of green production is shown in equation (12).

(12) $$F\left(z\right)=\frac{d\left(z\right)}{d\left(t\right)}=z\left(W_{1}-\bar{W}\right)=z\left(1-z\right)\left(W_{1}-W_{2}\right)=z\left(1-z\right)\left[x\left(G_{R2}+G_{s2}\right)+y\left(Q_{s1}-Q_{s2}+Q_{c1}\right)+\left(Q_{s2}-Q_{c1}-Q_{s3}+Q_{c2}\right)\right]$$

Hence, the system of replicated dynamic equations for the environmental governance game system are shown in equation (13).

(13) $$\left\{ \begin{array}{c} F\left(x\right)|\;=x\left(x-1\right)\left[yG_{s1}+z\left(G_{R2}+G_{s2}\right)-\left(G_{s4}+G_{c2}+G_{s2}+G_{s1}-G_{c1}-G_{R1}\right)\right]\\ F\left(y\right)|\;=yy(1-y\;)\left[xG_{s1}+z\left(D_{s1}+D_{c3}-D_{s2}\right)+D_{c2}-D_{c1}\right]\\ F\left(z\right)|\;=z(1-z\;)\left[x\left(G_{R2}+G_{s2}\right)+y\left(Q_{s1}-Q_{s2}+Q_{c1}\right)+\left(Q_{s2}-Q_{c1}-Q_{s3}+Q_{c2}\right)\right] \end{array}\right.$$

### 2.3 Stability analysis

According to evolutionary game theory, it is known that for a three-party game system, to explore its evolutionary stability, it is necessary to consider the stability of the boundary points of the game system, i.e., to consider the points that make the replicated dynamic equation system. Eight equilibrium points of the replicated dynamic equation system can be obtained, which are (0,0,0), (0,1,0), (0,0,1), (0,1,1), (1,0,0), (1,0,1), (1,1,0), (1,1,1). The Jacobi matrix is shown in equation (14).

(14) $$J=\left[\begin{array}{c} \frac{dF\left(x\right)}{dx}\;\frac{dF\left(x\right)}{dy}\;\frac{dF\left(x\right)}{dz}\\ \frac{dF\left(y\right)}{dx}\;\frac{dF\left(y\right)}{dy}\;\frac{dF\left(y\right)}{dz}\\ \frac{dF\left(z\right)}{dx}\;\frac{dF\left(z\right)}{dy}\;\frac{dF\left(z\right)}{dz} \end{array}\right]$$

The Jacobi matrix allows the eigenvalues of the above replicated system of dynamic equations, as shown in equation (15).

(15) $$\begin{array}{c} \lambda_{1}=\left(1-2x\right)[-yG_{s1}-z\left(G_{R2}+G_{s2}\right)+M]\\ \lambda_{2}=\left(1-2y\right)\left[xG_{s1}+z\left(D_{s1}+D_{c3}-D_{s2}\right)+D_{c2}-D_{c1}\right]\\ \lambda_{3}=\left(1-2z\right)[x\left(G_{R2}+G_{s2}\right)+y\left(Q_{s1}-Q_{s2}+Q_{c1}\right)+N] \end{array}$$

Where: $M=G_{s4}+G_{c2}+G_{s2}+G_{s1}-G_{c1}-G_{R1},N=Q_{s2}-Q_{c1}-Q_{s3}+Q_{c2}$, it can be obtained that the characteristic values corresponding to the above different equilibrium points, as shown in Table 3. Where $M=G_{s4}+G_{c2}+G_{s2}+G_{s1}-G_{c1}-G_{R1},N=Q_{s2}-Q_{c1}-Q_{s3}+Q_{c2}$, and $K=D_{s1}+D_{c3}+D_{c2}-D_{s2}-D_{c1},T=G_{R2}+G_{s2}+Q_{s2}-Q_{c1}-Q_{s3}+Q_{c2}$.

**Table 3:** Eigenvalues of equilibrium points.

Balancing Point	$\lambda_{1}$	$\lambda_{2}$	$\lambda_{3}$
(0,0,0)	M	$D_{c2}-D_{c1}$	N
(0,1,0)	$M-G_{s1}$	$D_{c1}-\;D_{c2}$	$Q_{s1}-Q_{s3}+Q_{c2}$
(0,0,1)	$M-G_{s2}-G_{R2}$	K	−N
(0,1,1)	$G_{s4}+G_{c2}-G_{c1}-G_{R1}-G_{R2}$	−K	$Q_{s3}-Q_{s1}-Q_{c2}$
(1,0,0)	−M	$G_{s1}+D_{c2}-D_{c1}$	T
(1,0,1)	$G_{s2}+G_{R2}-M$	$G_{s1}+K$	−T
(1,1,0)	$G_{s1}-M$	$D_{c1}-G_{s1}-D_{c2}$	$T-Q_{c1}$
(1,1,1)	$-\left(G_{s4}+G_{c2}-G_{c1}-G_{R1}-G_{R2}\right)$	$-\left(G_{s1}+K\right)$	$-\left(G_{R2}+G_{s2}+Q_{s1}-Q_{s3}+Q_{c2}\right)$

## 3 Analysis of simulation results

To analyze the equilibrium stability of a game system by means of a Jacobi matrix, it is necessary to ensure that the eigenvalues are all less than 0, i.e., $\lambda_{1}<0\text{,}\lambda_{2}<0\text{,}\lambda_{3}<0$, and three main scenarios are discussed below.

**Scenario 1:** The stabilization point is (0,0,0) when the central government adopts a conventional governance strategy for environmental management, leading to a relaxation of top-down transmission pressure. This scenario also results in an increased probability of collusion between local governments and polluting enterprises.

**Fig. 1:**
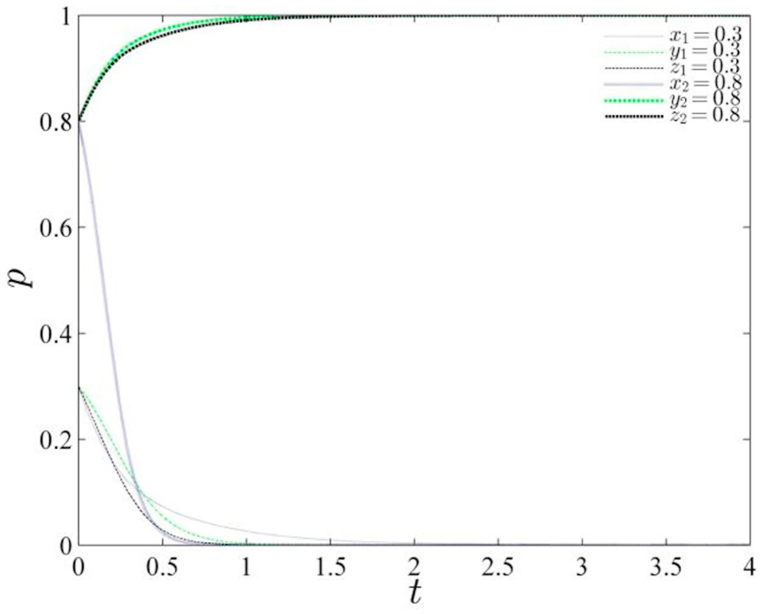
Evolutionary path of the gaming system under initial probability change.

Based on the parameter assignments detailed in Table 4, the study constructs a schematic diagram illustrating the evolutionary path for two distinct probability scenarios (refer to Fig. 1). Upon analysis, it is observed that the system converges to the equilibrium point (0,0,0) when the initial probability values are set at (0.3, 0.3, 0.3). However, an intriguing pattern emerges as the initial selection probability value is increased: the system gradually transitions from a state of equilibrium to an upward trend, signaling a departure from the stability of the central government's governance approach.

**Table 4:** Parameter assignment.

$G_{s1}$	$G_{R2}$	$G_{s2}$	$G_{s4}$	$G_{c2}$	$G_{c1}$	$G_{R1}$	$D_{s1}$	$D_{c3}$	$D_{s2}$	$D_{c2}$	$D_{c1}$	$Q_{s1}$	$Q_{s2}$	$Q_{c1}$	$Q_{s3}$	$Q_{c2}$
4	3	2	7	9	20	4	21	5	16	9	15	14	7	4	15	4

**Fig. 2:**
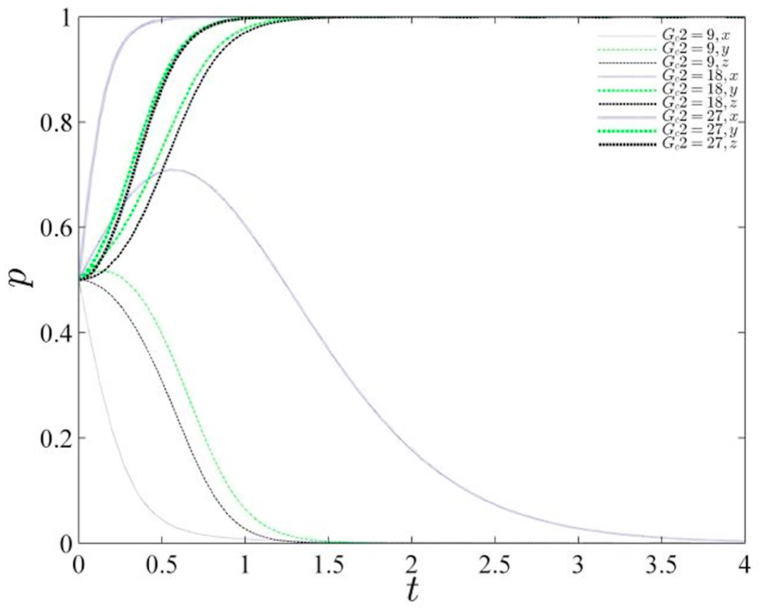
Diagram of the evolutionary path of the gaming system under changing reputation values of the central government.

The reputation influence factor, denoted as $G_{c2}$ assumes particular significance in the context of the central government's adoption of conventional governance, which is characterized by weakened supervision and subsequent reputational damage. This factor becomes pivotal in the comprehensive analysis of system stability throughout its evolutionary trajectory. To this end, the study explores the effects of $G_{c2}$ by assigning it values of 9, 18, and 27, respectively, and presents the corresponding evolutionary path diagram (refer to Fig. 2). The diagram provides compelling insights into the interplay between reputation damage and the governance paradigm. Upon close examination of the diagram, a discernible trend emerges: as the magnitude of reputational damage to the central government intensifies, there is a proactive shift from conventional governance towards an emphasis on environmental supervision. This transition signifies a strategic response to the escalating reputational challenges faced by the central government, wherein the imperative to prioritize environmental concerns becomes increasingly pronounced in the face of heightened reputation damage.

**Scenario 2:** In the scenario where the equilibrium point is (1,1,1), the central government prioritizes environmental governance and implements a variety of measures, including environmental supervision, to advance environmental management. Concurrently, local governments take proactive steps to address environmental pollution issues, while polluting enterprises adopt green production strategies.

**Fig. 3:**
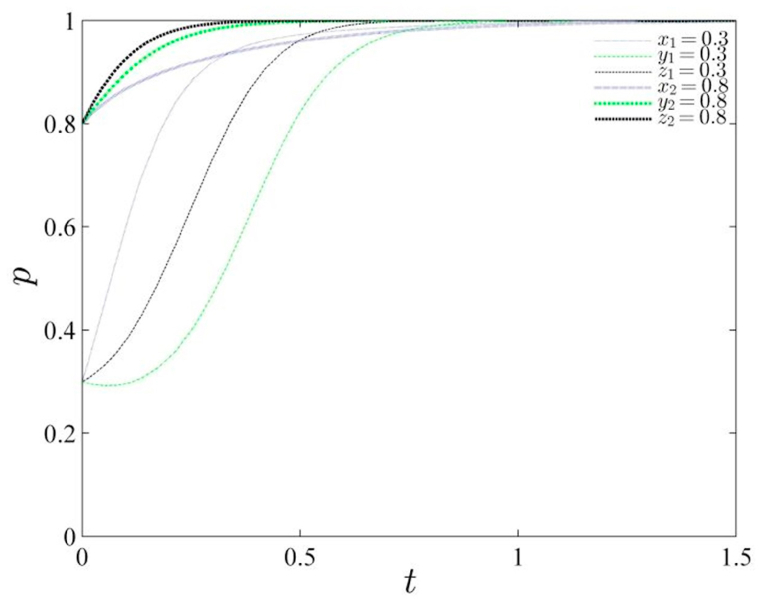
Diagram of the evolutionary path of the gaming system under the initial probability change.

Based on the assigned parameter values detailed in Table 5, a schematic diagram illustrating the evolutionary trajectory under two different probability scenarios provides intriguing insights (refer to Fig. 3). When the initial probabilities are set to (0.3, 0.3, 0.3), the system converges towards the equilibrium point (1, 1, 1). As the initial probabilities increase, the stable strategy of the entire system remains unchanged, albeit with a slower convergence rate towards 1. This indicates that, under the given parameter values, simply altering the initial probabilities does not significantly impact the system's stability trend. This scenario reflects a common situation where active government involvement and guidance are necessary, along with the formulation of rational policy measures. Simultaneously, polluting enterprises, considering both profitability and cost considerations, make informed decisions. Furthermore, it suggests that increasing punitive costs within the regulatory policy framework can effectively encourage compliance strategies among enterprises and local governments.

**Table 5:** Parameter assignment.

$G_{s1}$	$G_{R2}$	$G_{s2}$	$G_{s4}$	$G_{c2}$	$G_{c1}$	$G_{R1}$	$D_{s1}$	$D_{c3}$	$D_{s2}$	$D_{c2}$	$D_{c1}$	$Q_{s1}$	$Q_{s2}$	$Q_{c1}$	$Q_{s3}$	$Q_{c2}$
8	3	7	8	15	11	6	22	5	15	8	14	13	10	4	13	4

**Fig. 4:**
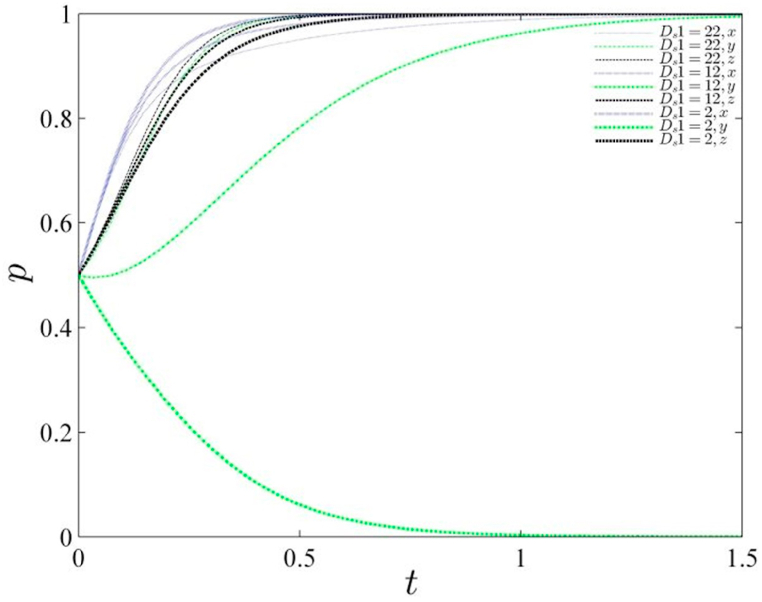
Evolutionary path of the gaming system under changing reputation values of local governments.

The study introduces $D_{s1}$ as the reputational influences of the local governments, representing the reputational gains that local governments can obtain when they are actively involved in governance. The study analyzes the changes in system stability as $D_{s1}$ varies, assuming values of 22, 12, and 2 respectively, and presents the evolutionary path diagram below (refer to Fig. 4). Analysis of the evolutionary trajectory reveals that as the reputation return value of local governments gradually decreases, significant changes occur in their strategies. Initially, the stability stabilizes at 1, then decreases for a period before converging back to 1, and finally rapidly converges to 0 when the reputation value reaches 2. This demonstrates the sensitivity of reputation value to the strategies employed by local governments. If the reputation value becomes too low, it directly affects the government's credibility. Therefore, it is crucial for local governments to prudently maintain their own credibility and choose a certain level of active governance.

**Scenario 3:** In the case where the stabilization point is (0,1,1), the central government tends to adopt the strategy of conventional governance, while the local governments engage in active governance and motivate polluting enterprises to engage in green production.

**Fig. 5:**
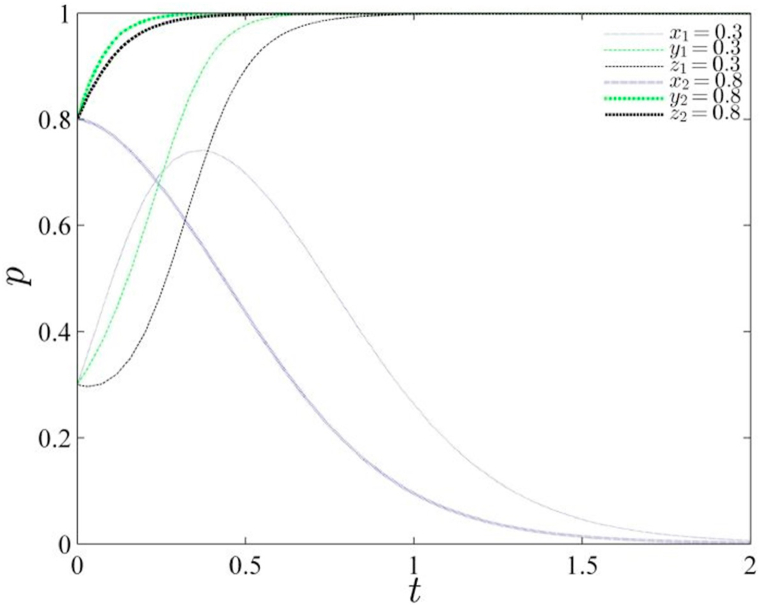
Schematic diagram of the evolutionary path of the gaming system under the initial probability change.

Considering the assigned parameter values detailed in Table 6, the study constructs a schematic diagram that delineates the evolutionary trajectory under two distinct probability scenarios (refer to Fig. 5). When the initial probability is set to (0.3, 0.3, 0.3), the system converges to the equilibrium point (0, 1, 1). Notably, as the initial probability increases, the overall stability strategy of the system remains unaltered, yet the central government converges to 0 at an accelerated rate in a linear fashion. Conversely, when both initial probabilities are at 0.3, the central government undergoes a phase of linear escalation. This phase is characterized by less stringent regulation by the central government, with considerations for potential rebounds by local governments and enterprises, resulting in continued regulation for a specific duration before gradually converging to 0. However, when the initial probability is elevated to 0.8, local governments and enterprises demonstrate an entrenched proclivity toward their established strategies from the preceding period. Consequently, they persist in active governance and the promotion of green production. In response, the central government opts for conventional governance, diminishing government investment and maximizing social returns.

**Table 6:** Parameter assignment.

$G_{s1}$	$G_{R2}$	$G_{s2}$	$G_{s4}$	$G_{c2}$	$G_{c1}$	$G_{R1}$	$D_{s1}$	$D_{c3}$	$D_{s2}$	$D_{c2}$	$D_{c1}$	$Q_{s1}$	$Q_{s2}$	$Q_{c1}$	$Q_{s3}$	$Q_{c2}$
8	3	7	10	15	20	6	20	5	15	8	9	12	10	8	13	4

**Fig. 6:**
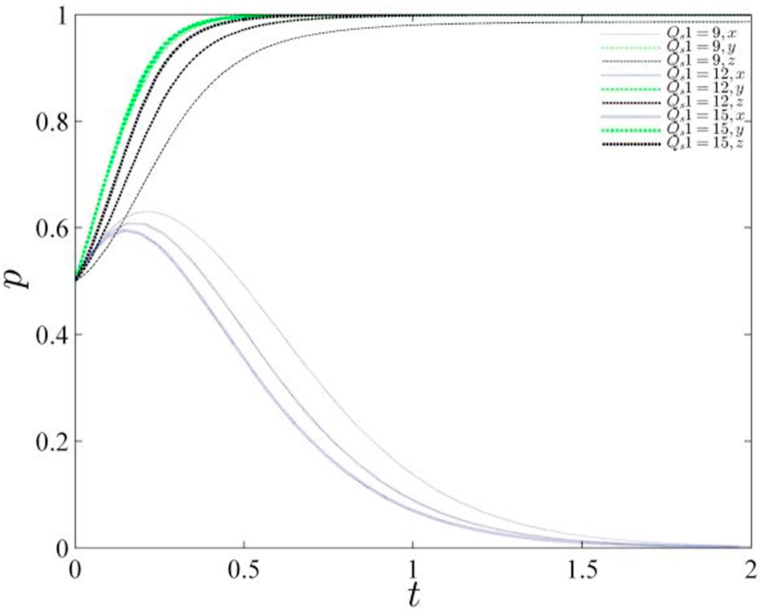
Evolutionary path of the gaming system under changing corporate reputation values.

Further analysis involving the variation of $Q_{s1}$, an influential factor on enterprise reputation (representing the reputation value generated by a polluting enterprise choosing green production), assumes values of 9, 12, and 15 respectively. The corresponding evolutionary path diagram is depicted below (refer to Fig. 6). Upon analyzing Fig. 6, it becomes apparent that the ultimate strategy of the central government is inclined towards choosing conventional governance. Nevertheless, as the reputation value of local enterprises escalates, the time period for the central government to converge to zero decreases. This phenomenon arises from the accelerated convergence of the enterprise itself to the value of 1, which weakens the regulatory influence of the central government. Consequently, the central government converges to zero within a shorter timeframe. Notably, a higher reputation value for the enterprise expedites its convergence to 1 due to the potential for increased profits. Faced with augmented net profits, enterprises accelerate the pace of green production, thereby elucidating the direct correlation between enterprise reputation and the adoption of sustainable practices.

## 4 Discussion and conclusion

The central system for environmental protection inspection constitutes a formidable tool in the realm of environmental management, serving as a pivotal cornerstone for endeavors to construct an ecological civilization. This study constructs a tripartite evolutionary game model of environmental governance in China, grounded in the perspective of the relationship of government-enterprise. It evaluates the marginal effects of the environmental protection mechanism under both conventional and mobilized governance approaches, as well as the collusive behavior between government and enterprises vis-à-vis the mechanism. The model offers a robust and insightful framework for analysing environmental governance dynamics, with the potential to inform policy interventions and contribute to the advancement of sustainable environmental governance practices.

In order to investigate the role of reputation signals in environmental governance, the study incorporates a reputation mechanism into the evolutionary game analysis. The findings demonstrate that the evolutionary game system of environmental governance accords great value to the reputation mechanism, exerting a significant influence on governmental decision-making. Policies wield considerable impact on the strategies pursued by polluting enterprises, with higher penalties under supervisory regulations directing enterprises toward compliance strategies. Moreover, the local governments typically employ the strategy of active governance while purposely avoiding instances of collusion with enterprises due to the sensitivity of reputation signals to environmental governance strategies, which could directly undermine the local government's credibility. The central system for environmental protection inspection functions as a tool for environmental governance rather than its ultimate aim. The devolution of environmental governance pressure from the central level of government to the local level can assist local governments in cultivating a green development mindset and guiding enterprises to modify their production processes, thus paving the way for the eventual realization of the symbiotic growth of economic and ecological civilization.

Compared with previous studies, this study has identified the pivotal role played by the reputation mechanism in environmental governance, a conclusion that aligns with the findings of existing scholarship. For instance, numerous previous studies have established that the reputation mechanism represents an effective tool for environmental governance [28,29], while some research has further demonstrated that the implementation of environmental regulations can bolster enterprise compliance by enhancing credibility [15,30]. Similarly, several studies have indicated that the implementation of environmental taxes can incentivize enterprises to adopt more environmentally-friendly practices by increasing their pollution costs [[31], [32], [33], [34]]. Nonetheless, our study delves deeper into the influence exerted by the reputation mechanism on governments, enterprises, and the general public, providing concrete viewpoints and recommendations for tackling the issue of government-enterprise collusion.

Moreover, our study underscores the significance of the reputation mechanism in environmental governance, whereas previous research has primarily focused on specific environmental governance mechanisms within particular sectors. Our study sheds light on the global mechanism of an environmental governance game. We posit that the central system for environmental protection inspection serves as a means of environmental governance rather than an end in itself. This differs from prior research, which viewed the central inspection system as an objective-oriented environmental governance tool directly impacting governmental and corporate behaviors [12,16,35]. Conversely, our study asserts that the primary objective of the central system for environmental protection inspection is to facilitate local governments' adoption of a green development perspective, encourage citizen participation in environmental protection and the cultivation of environmental knowledge, guide enterprises toward eco-friendly production methods, and promote coordinated economic and ecological development. This perspective constitutes another distinctive contribution of our study. The divergence between our findings and earlier research may be attributed to variations in the research subjects and methodologies. For instance, this study examines the evolutionary game system of environmental governance, while previous studies may have concentrated on single-stage environmental governance mechanisms or the environmental practices of specific industries. By employing a system dynamics model, this study comprehensively considers the interactions and feedback mechanisms among various factors, unveiling the complexity and diversity inherent in the evolutionary game system of environmental governance. Previous studies may have relied on statistical analysis or empirical research techniques, which could limit their ability to fully grasp the overall characteristics of the evolutionary game system of environmental governance.

In conclusion, this study contributes fresh perspectives and potential solutions that can significantly inform future research and practice in the field of environmental governance. The incorporation of a reputation mechanism into the evolutionary game model presents a novel avenue for further exploration and verification. To advance the understanding in this domain, future research should delve into the specific mechanisms of the reputation mechanism in environmental governance and investigate its intricate impact on government decision-making and environmental governance strategies. Moreover, exploring the relationship between the central system for environmental protection inspection and local government green development is imperative for gaining deeper insights into China's environmental governance practices. Such endeavors will not only enrich the understanding of environmental governance in China but also provide valuable references for environmental governance in other countries and regions. By delving into these areas, researchers can contribute to the development of more effective and sustainable environmental governance practices worldwide.

## Funding

This work was supported by the Humanities and Social Sciences Foundation of the 10.13039/501100002338Ministry of Education of China (Grant Number: 23YJC840022); the Social Science Foundation of 10.13039/501100002949Jiangsu Province (Grant Number: 23SHC009); the General Project of Philosophy and Social Science Research in Universities in 10.13039/501100002949Jiangsu Province (Grant Number: 2023SJYB0243); the Research Starting Foundation for Talented Scholars of 10.13039/100008964Nanjing Normal University (Grant Number: 184080H202A177).

## Data availability statement

Data included in article/supp. material/referenced in article.

## Ethical statement

This work was approved by Ethics Committee of Nanjing Normal University, with the approval number: 184080H202A177. All procedures performed in this study were in accordance with the ethical standards of the institutional and national research committee and with the 1964 Helsinki Declaration and its later amendments or comparable ethical standards.

## Informed consent statement

This article does not contain any studies with human participants performed by any of the authors.

## Additional information

No additional information is available for this paper.

## CRediT authorship contribution statement

**Feng Cao:** Writing – original draft, Software, Methodology, Conceptualization. **Ling Zhang:** Validation, Funding acquisition, Conceptualization. **Weiyun Wu:** Writing – original draft. **Sijia Han:** Writing – original draft. **Zhaocheng Wu:** Writing – review & editing, Writing – original draft, Software. **Yihan Wu:** Writing – review & editing, Validation, Funding acquisition.

## Declaration of competing interest

The authors declare that they have no known competing financial interests or personal relationships that could have appeared to influence the work reported in this paper.
